# Experiences in child and adolescent psychiatry training: an international qualitative study

**DOI:** 10.1186/s13034-025-00871-y

**Published:** 2025-04-05

**Authors:** Peter Deschamps, Brian Jacobs, Anna Sofie Hansen, Tjhin Wiguna, Suaad Moussa, Aisha Sanober Chachar, André Luiz Schuh Teixeira da Rosa, Víctor Pereira-Sánchez, Marie-Aude Piot

**Affiliations:** 1 Karakter, Academic Centre for Child and Adolescent Psychiatry, Nijmegen, The Netherlands; 2 Institute of Psychiatry, Psychology and Neuroscience, South London and Maudsley NHS Foundation Trust, London, UK; 3 Psykiatrien- Region Nordjylland, Aalborg, Denmark; 4 Faculty of Medicine, Universitas Indonesia - dr. Cipto Mangunkusumo General Hospital, Jakarta, Indonesia; 5 Kasr Al Ainy Medical School, Cairo University, Giza, Egypt; 6 Synapse Pakistan Neuroscience Institute, Psychiatry, Aga Khan University, Karachi, Pakistan; 7 Federal University of Rio Grande do Sul and Hospital de Clínicas de Porto Alegre, Porto Alegre, Brazil; 8 Department of Child and Adolescent Psychiatry, NYU Grossman School of Medicine, New York, USA; 9 Department of Child and Adolescent Psychiatry, Université de Paris-Cité, Necker-Enfants Malades Hospital, Paris-Saclay University, Paris, France

**Keywords:** Internship, Residency, Mentorship, Internationality, Qualitative research

## Abstract

**Background:**

Experiences of medical specialist trainees in psychiatry can be informative for those who seek to improve post-graduate training. This study aimed to explore the experience of child and adolescent psychiatry (CAP) trainees across different training settings and cultures worldwide to seek out similarities and differences.

**Methods:**

A convenience sample of CAP-trainees (n = 36) and -trainers (n = 54) was recruited internationally. All provided a narrative account on aspects of training in their region (n = 27 different countries), either first-person (trainees) or through external perception (trainers). Thematic analysis was used for inductive treatment of the data.

**Results:**

Trainees’ and trainers’ perspectives covered psychological security of the educational framework (including a comprehensive curriculum; social support and recognition of local context), specific skills to be acquired during training and the effects of the social-cultural dimension on mental health (training). Trainers’ perspectives highlighted the importance of support and training for supervisors; an overall view to make sense of the training framework; and of looking at training through objective and subjective frameworks of thinking to understand and guide trainees’ pathways.

**Conclusions:**

Experiences from international psychiatry trainees suggest themes that may guide further development of international standards in psychiatry specialist training on a tailored and consistent supervision framework for trainees. For their trainers, a learning community may offer ongoing support, supervision skill development and help reflect on overall views on systems of care.

## Background

Psychiatric practice, like all other medical disciplines, is partly based on common international scientific knowledge and a base of evidence that has grown over the past decades. However, psychiatry, perhaps more than many medical specialties, is also embodied in a local and cultural context. Psychiatric service provision varies substantially across nations and regions. These variations are a result of socioeconomic circumstances as well as cultural differences. In some countries, CAP service provision and training is organized as an independent medical specialty, in others it is a sub-specialty of general psychiatry and in others there is little or no CAP service provision yet. This study aimed to explore the experiences of child and adolescent psychiatry (CAP) trainees worldwide as a basis of reflection on the similarities and differences in training practices.

The development of evidence-informed medicine in (child) psychiatric practice benefits from international guidelines that have become increasingly available since the nineteen-eighties. Several series of clinical practice guidelines have become internationally accessible for trainees and clinicians together with international textbooks (e.g., the AACAP practice parameters and IACAPAP e-textbook of child and adolescent mental health) [1, 2]. Common ground for the use of these guidelines in CAP training globally comes from the finding that mental disorders have similar incidence rates worldwide and that the heterogeneity in estimates may well not be a function of geographic location but rather from sample representatives and diagnostic methods [3]. Although only 10% of treatment trials come from low- and middle-income countries, the limited evidence from these studies points toward similar effects of common interventions for (child) mental health across the globe [4]. Most treatment can be adjusted pragmatically to socioeconomic circumstances and work with religious and social support specific to each culture to help recovery [7]. On the other hand, both socioeconomic and cultural differences may limit the applicability of evidence-informed practices in some regions.

Training practices follow current practices and tend to shape future clinical practice. Thus, similarities and differences in the applicability of the international base of evidence are likely to be reflected in training practices. Over the past few decades, international groups of trainers have started to combine experience from several regions of the world to establish a consensus on joint curriculum frameworks for the essential competences required for trainees [8]. These frameworks describe *what* knowledge (content of training) is transferred to the next generation of child and adolescent psychiatrists. There have also been some efforts to describe similarities and differences in training methods and systems and in *how* knowledge is transferred. The evidence for how to best train these competencies is still limited, as most studies on training itself have been descriptive in nature [9], and only a few studies have been conducted exploring what training practices in psychiatry may work for whom in what circumstances. Some have focused on specific training packages, such as simulation-based education [10] or problem-based learning [11]. Others have focused on themes, e.g., addictions [12], family therapy [13], and trainees’ well-being [14].

Most approaches to international training similarities and differences have involved descriptive, quantitative surveys. They report *what* the international differences *are*. They do not explore the qualitative experience of trainees and trainers. Doing so may help us understand *why* these differences exist and what we can learn from them. A qualitative approach allows the trainer to better ‘put themselves in the shoes’ of trainees’ experiences. This qualitative approach for training in child psychiatry has recently been applied to undergraduate medical students. The first encounters with adolescents with mental disorders proved to be highly challenging for young medical students who have just (or not yet) completed their own adolescent development [15]. A small feasibility pilot study of postgraduate trainees showed that it was possible to survey the experiences of CAP trainees and trainers internationally, using online recruitment and open questions sufficiently broad to allow qualitative thematic analysis of their answers. The present study builds upon that feasibility pilot in a larger and more internationally diverse sample [16]. To provide a better context for interpretation of the results, indicators of the quality of the training system were explored. The main analysis tested whether the overall themes that arose during the pilot study could be reproduced and refined in the present sample.

## Methods

### Research design

In an online survey, demographics of each respondent (i.e., country, trainee or trainer) were first collected to provide background information for the main qualitative analysis. A brief survey explored two indicators of the quality of the training system by asking participants’ general estimates on a scale of 1–10: (a) how well they perceived the system prepares trainees in CAP for their role as medical specialists both in the short and the long term and (b) how well they trusted the quality of care provided by trainees.

Thematic analysis (TA) was chosen as the qualitative approach to explore and identify patterns of meaning within narrative data, emphasizing the most salient clusters of content [17, 18]. This method is often used to investigate a group’s conceptualization of a specific phenomenon [19]. As TA offers flexibility regarding the framework of thinking, we adopted an approach, best suited to explore experiences and meaning in a straightforward way, assuming a more unidirectional relationship between meaning, experience and language [20, 21]. This is also known as an essentialist approach.

To explore the similarities and differences in training practices related to local, societal and cultural differences, a description of the experience of training in various countries was sought. These experiences were obtained via a survey from the perspective of trainees and experienced trainers, including those concerned with or responsible for the organization of training in their country.

### Participants

The participants were approached as a convenience sample through a group of international child psychiatry trainers that were gathered around medical specialization training practices from an international perspective. The working group consisted of a combination of training experts and trainees involved in (inter)national training organizations. Through them, we aimed to recruit a sample with a wide variation in region and role (trainee and trainer). This double perspective sought to provide complementary views, as well as information on agreements and potential discrepancies between these different perspectives. The aim and modalities of the study were described to the participants, and permission to use the data for the research was obtained.

### Data collection

The working group members each invited connections in their network to contribute to the study. In an online survey, we asked for a contribution to describe two different levels of subjective experience and perspective: the trainee and the trainer. The main goal was to provide their experience and perspective. Some open-ended questions were designed by four of the working group members after several discussions (see Table 1). These were preceded by a short questionnaire about child psychiatry training conditions in the participant’s country of origin to help interpret subjective experiences and perspectives (see Table 1).

**Table 1:** Questions designed to prompt reflexivity and personal perspectives from trainees and trainers

Demographic	
Which country's child and adolescent psychiatry (CAP) training are you providing information about?	Text
From what position are you answering the questions about CAP training?	Check boxes (trainee, trainer/supervisor, other)
Indicators of perceived quality of the training system	
How well you think the system trains CAPS for the role that they will occupy now as specialists? (1 being not at all, and 10 as well as possible)	Scale between 1–10
How well you think the system trains CAPS for the role that they will occupy in the future as specialists? (1 being not at all, 10 being as well possible)	Scale between 1–10
If your child would need psychiatric care, to what extend would you leave your child with a CAP resident/trainee? (1 = not at all, 10 = I absolutely would)	Scale between 1–10
Subjective experience and perspective	
For trainees	Free text
Please provide your experience and perspective on training within CAP in your country	
What is it like to be a trainee in your country? What do you enjoy and appreciate most and what are the challenges and things you would like to change? Are there features to your training that you think are specific to the context you are in (cultural, geographical, resource specific, etc.)? Anything else, you find relevant to share	
For trainers	Free text
Please provide your experience and perspective on training within CAP in your country	
What is it like to be a trainer/supervisor in your country? What do you enjoy and appreciate most and what are the challenges and things you would like to change? Are there features to your training that you think are specific to the context you are in (cultural, geographical, resource specific, etc.)? Anything else, you find relevant to share	

Thematic analysis has no sample size constraints because the wealth of data is situated in the richness of participants’ narratives and not in the number of participants [18]. Our aim was to perform a global exploration with substantial variation in cultural circumstances, rather than to survey all regions of the world uniformly or to cover all countries. The researcher group estimated that data sufficiency was achieved, that is, to reach an in-depth understanding sufficient to provide initial insights into patterns [22].

### Analysis

For the contextual data, continuous variables are presented as medians and interquartile ranges (non-normal data distribution), and differences between groups (trainers versus trainees) were tested via the Kruskal‒Wallis equality-of-populations rank test.

Qualitative data written reports were analyzed inductively by one of the authors, on the basis of Braun and Clarke’s 6-stage thematic analysis method [18]. Iterative analysis was conducted throughout the data collection. Through thematic analysis, the authors sought systematic identification, interpretation and development of themes derived from the content of the data. The two perspectives—those of trainees and trainers—were first treated separately and given the substantial overlap between both perspectives, combined.

The first stage was devoted to reading and rereading the written reports. At stage two, two authors independently conducted line-by-line coding. They searched for common themes of meaning between respondents, an inductive and recursive process, while using constant comparisons within and between accounts. Then, the themes were combined to reflect major features and patterns in the data during Stage 3. The next two stages were devoted to reviewing all the themes and coding with the research team to ensure consistency while refining the relevance of the themes. This enabled the discussion of alternative interpretations until a consensus on the pattern was reached. The final stage closed the analytic process by completing the written report.

The derived themes and subthemes could not be submitted to participants for their comments to verify that this reflected their experience because their participation was anonymous.

### Reflexivity

Qualitative research aims at reporting subjective reality with the greatest rigor and validity. This process is improved by “triangulation” process, that is confrontation and enrichment by several views on data collected. This helps to limit influence of researcher personal view on the research while triggering reflexivity. Thus, our research team intentionally included researchers from various regions, different gender, age-group and professional experience in CAP. “

One researcher (MAP) is a CAP trainer who recently completed her PhD in psychiatric education. She has been strongly involved in undergraduate and postgraduate teaching in mental health at her university while being involved in CAP training daily through clinical teaching. She focused on following the process and step methods. Using notes and memos she assessed her prior assumptions regarding the research phenomenon. She was supported through several research meetings and exchanges with other international researchers, providing triangulation of the qualitative findings.

The other researchers all held active positions within the Bureau of the CAP section of the Union European of Medical Specialists (UEMS-CAP). Two of the authors (PKHD from the Netherlands, who holds a PhD and a Teaching Scholarship from Utrecht University, and BJ from the UK, past-president of the Board of Education of UEMS-CAP) had experience as CAPs in clinical work, teaching, and supervision and in the organization of local, national, and international training activities as training program directors. The other author ASH from Denmark was a trainee in her second half of CAP training. She had previously also held a role in the European Federation of Psychiatric Trainees (EFPT). Thus, they were able to reflect on the results of the study by integrating their personal experience of being a CAP trainee earlier on in their careers, of being a trainer in their own region and of previous exchange of ideas with colleagues from other regions.

For the present study, the three who had previously worked and published together on international cooperation in CAP training served as secondary readers of the themes that were initially analyzed and presented by MAP. None of the authors participated in the data collection by providing narratives of themselves.

Electronic anonymization of the data collection ensured that there was no previous relationship between researchers and participants, which could have influenced the process of researchers’ analysis, for example, creating a risk for trainee participants that their participation might influence their educational pathway.

### Ethical agreement

The French Institutional Review Board of “CERAPHP” (“Comité d'éthique de la Recherche AP-HP Centre”; that is, “Ethical Research Committee of Public Health Assistance of Paris Center”) provided permission within the reference “2022-07-01”.

## Results

Ninety contributions of training experience were received: 36 from trainees and 54 from trainers. The countries of origin (n = 27) of the participants are reported in Table 2.

**Table 2:** Participants’ countries of origin

Region	Countries	Trainees N = 36	Trainers N = 54	Total N = 90
Europe	Albania Austria Denmark Estonia France Greece Hungary Portugal Spain Slovenia Switzerland The Netherlands United Kingdom	21	18	39
North-America	Canada United States of America	7	4	11
South-America	Argentina Colombia	1	1	2
Asia	Indonesia Japan Kingdom of Bahrain Pakistan Sri Lanka United Arabic Emirates	3	5	8
Africa	Algeria Egypt	2	3	5
Oceania	Australia New Zealand	2	23	25

The indicator of perceived quality of training, defined as preparation for the role of medical specialist in child and adolescent psychiatry, had overall median scores of 7.9 (trainees) and 7.8 (trainers) for current performance on a scale between 1 and 10. A similar overall median score was found for future performance scores of 7.6 (trainees) and 7.4 (trainers). The indicator defined as trust in the referral of a loved one had overall median scores of 7.5 (trainees) and 7.6 (trainers). No statistically significant differences were found between trainers and trainees. All variables showed substantial variation, with an interquartile range toward the lower end of the scale of one point or more and an interquartile range toward the higher end of the scale of half a point or more (Table 3).

**Table 3:** Indicators of perceived quality of training

	Trainees		Trainers		P-value
	Median	(IQR)	Median	(IQR)	
How well do you think the system trains CAPS for the *current* role as specialists?	7.9	(6.2–8.7)	7.7	(6.3–8.2)	0.42
How well do you think the system trains CAPS for the *future* role as specialists?	7.6	(6.5–8.6)	7.4	(6.2–8.4)	0.40
Would you trust the psychiatric care of your own child to a CAP resident/trainee?	7.5	(6.2–8.7)	7.6	(5.0–8.7)	0.49

**Fig. 1:**
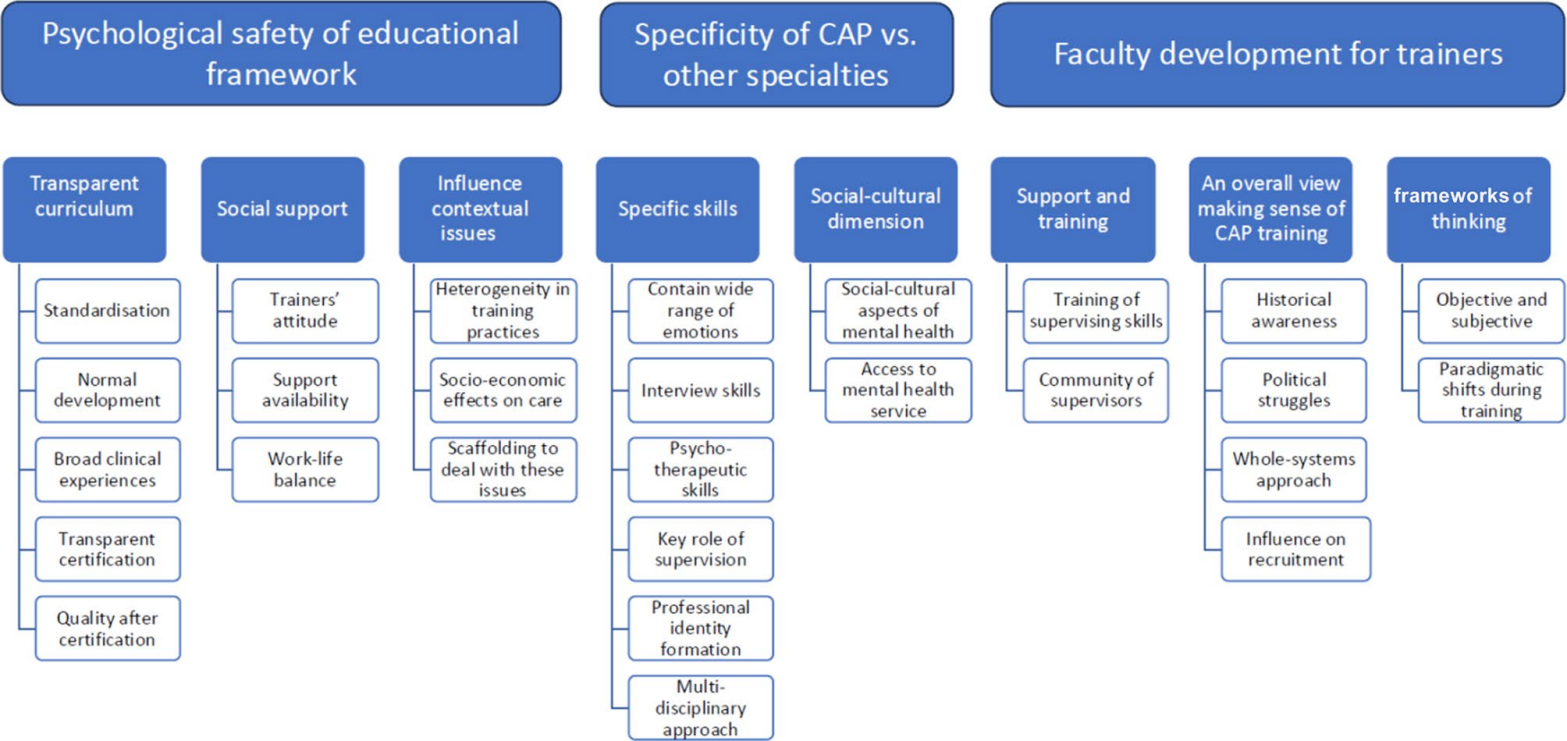
Summary of themes and sub-themes related to CAP training based on international experiences of both trainees and trainers combined

Both trainees and trainers emphasized two main themes: the psychological security of the educational framework and the specificities of CAP training compared with other medical specialties. Figure 1 summarizes the themes and subthemes. Trainers emphasized an additional main theme regarding faculty development in training competencies. Figure 1 provides an overview, and Tables 4, 5 and 6 provide more details and examples of quotations.

### Theme 1: Psychological security of the educational framework

**Table 4:** Psychological safety of educational framework for trainees

	Trainees’ quotations	Trainers’ quotations
Comprehensive and transparent curriculum		
Curriculum with high level of structure and standardisation	“Being a trainee in my country is exciting because the place where I am studying has a structured educational curriculum.” (Asia)	
Strong knowledge-basis on normal development	“I appreciate being able to learn about typical development prior to taking care of children with psychopathology.” (North-America)	“It is crucial that CAPs are trained to have a deep understanding of ‘normal’ child development, including brain and cognitive development, family development across the lifecycle, developmental trauma…” (Oceania)
Broad enough range of clinical experiences (age and setting)	“… the specialty curriculum is wide enough, comprising lots of both hospital and ambulatory psychiatry settings, as well as other interrelated professional fields (pediatrics, neurology, social work, special education, psychology…). (Europe)	“I have tried to provide a range of experiences for the trainees e.g., working as co-therapist with a range of clinicians in the team, opportunities to prescribe, to deliver individual therapy while also being involved in family therapy… meetings to learn about the roles and responsibilities of consultants,…” (Oceania)
Transparent process of certification	“There seem to be bureaucratic processes that might make trainees seem'infantilised'—yet on reflection, there needs to be a system of assessment—both summative and formative.” (Europe)	
Maintaining quality after certification		“There is no indicator to properly evaluate the quality of physicians. Almost all psychiatrists do not disclose their treatment outcomes” (Asia)
Social support		
Trainer’s attitude	“What I appreciate most is the safe training climate (…). There is an open atmosphere, in which contact with supervisor/trainer is easily accessible.” (Europe)	“It is a balance between compassion with the trainees and consideration of their boundaries, and on the other hand encouragement to go beyond their limits and drawing lines to keep them up to the demands of the traineeship.”. “I try to… generate qualified professionals with self-esteem.” (Europe)
Support availability	“Challenges include the lack of certified (and uncertified, but trained) faculty in university hospitals, and the insufficiency of CAP service infrastructure in many places—all of what would make it difficult to host quality training programs in many hospitals, and to ensure a right balance between self-direction and always having senior consultant support.” (Europe)	
A good work-life balance	“It was a burden on my social and familial life.” (Africa)	“Having their own children and the interaction with them often amplify the struggle of their own emotional processes and a new adequate work-life balance has to be found.” (Europe)
Recognition of influence of contextual issues		
A perception of high heterogeneity in training practices	“The experience of being a CAP trainee in Portugal really depends on the hospital in which you work. (…) Because of the lack of universal prevention programs and bad articulation with community structures, as well as millions of Portuguese people lacking a family doctor” (Europe)	“I think the main challenge to organize a national training program is to keep an equilibrium between harmonization of practices and a degree of local freedom.” (Europe)
Heterogeneity in care-provision related to social and economic context	“Despite being trained in a skillful way to perform as evidence-based and resource-aware therapists and clinicians, our current healthcare-system does not accommodate very well to the state-of-the-art treatments that we often learn in the program.” (South-America)	“After 20 years, none of us worked anymore for the public health system, as the salary was low and burn out was high. All of us changed careers to more fulfilling and financially rewarding ones!” (Europe)
Scaffolding to deal with socio-economic issues for trainees	“Well, I can say that being a trainee for child and adolescent in Albania has a lot of difficulties. First of all, you don't get paid during your residency and this make it really hard in the economic point of view.” (Europe)	“Some of the challenges we face [for CAP training in our country] are… the unsatisfactory quality of the delivered services due to workload and limited… services… shortage of medications and… digital system.” (Egypt)

Trainee: “The right balance between self-direction and always having senior consultant support.”

Trainer: “It is a balance between compassion with the trainees and consideration of their boundaries, and on the other hand encouragement to help them to go beyond their limits and… to the demands of the traineeship.”

The first subtheme covered the need for *a comprehensive and transparent curriculum*. A sufficient degree of standardization of the curriculum appeared to be a factor that provided a safe structure in training, while its absence aroused feelings of being overwhelmed and of exhaustion in trainees, which may inhibit learning. There was a need to first have a strong base of knowledge about normal development before learning about CAP conditions in a broad enough range of experiences, comprising various clinical settings within CAP and in adjacent fields. Trainers perceived the need for a sufficiently wide curriculum in both practical and theoretical ways. They thought it should offer a broad range of experiences to develop safe patient care and to help them practise in different settings.The necessity of some kind of summative assessment and examination was recognized. Some trainees regretted the absence of certification goals and requirements in their country. In their view, the high level of motivation and skills necessary to work with children and adolescents was left unrecognized. Others criticized the many issues that needed justification in the certification process and found that these issues created feelings of ‘infantilization’. Some trainees noted there were more training positions than available candidates. They were concerned that this would lead to lowering the bar and decreasing the overall quality of training programs. For both continuing medical education and quality assurance, some trainers thought that the systems were inadequate in their countries.

The second subtheme concerned *social support*. Both trainers and trainees appreciate a benevolent and less hierarchical attitude. This is seen as a strong factor supporting the ability to learn from mistakes and allows for a smooth transformation from trainee to medical specialist. Some trainee participants highlighted the subtlety of appropriate mentorship that that is tailored to individual trainees’ needs. This should be in line with their stage of training from a mentor who does not hold a position of formal evaluation or power over the trainee. Support embedded in a positive learning relationship was appreciated. Trainers emphasized a safe training environment implying well-adjusted trainer support tailored to the trainee’s needs as essential to support self-confidence and self-efficacy in trainees. An appropriate work‒life balance with good enough relationships with loved ones was also mentioned as an important condition for efficient learning during training. The trainees mentioned a negative spiral interaction. Adverse learning conditions (e.g., overwhelming clinical work, the tremendous effort and time required to reach learning goals) all put stress on their time and mental availability for personal relationships. Limited opportunity for social support further decreases the ability to deal with stressful situations. High stress reduces their mental availability for skills learning, especially the ability to contain a wide range of emotions and psychotherapeutic skills essential to manage difficult clinical situations. As a result, some trainees reported increased conflict in their personal life. Some trainers drew particular attention to their trainees’ struggling with their own roles as parents and of the importance of a good balance at home as well as at work.

Finally, the educational framework can help trainees recognize and adjust to the *influence of the socioeconomic and political context*. Some trainees noted the lack of well-trained health workers in mental health in their country overall or in specific (rural) areas. In other places, cultural or religious aspects limited access to the mental health care system. Other trainees noted a gap between the evidence-based practice promoted in training, the current limits of healthcare provision in their country and the need to discuss this during training with their supervisors. Most trainers were aware of geographical heterogeneity in CAP training and service provision across countries. For example, in a South-American country, training was still closely linked with psychoanalysis, with less focus on evidence-based interventions and scientific research. Trainers from Oceania highlighted that working in an isolated area allowed trainees to develop skills to work with indigenous youth and manage severe disorders with little resources but made them miss out on other approaches, e.g., family work in community clinics. Clinical placements were noted to be difficult to accommodate because services are so stretched, and a stark difference was noted between larger centers’ service provisions and rural settings. In a European country, there were concerns about the work force turning away from public health care or leaving the country because of low salaries in CAP.

### Theme 2: Specificity of CAP training content in comparison with other medical specialties

Trainee: “Since psychotherapy has a primary role among the therapeutic tools in child and adolescent psychiatry, I think that much more emphasis should be placed on teaching psychotherapy”.

Trainer: “Child and adolescent psychiatry may draw on a different set of clinical principles…, i.e., in being more oriented to systems, relationships, and development.”

**Table 5:** Specificity of CAP training versus other specialties

	Trainees quotations	Trainers quotations
Specific skills		
Ability to contain a wide range of emotions	“…context were difficult… it also taught me a lot—how to prioritize, (…) evaluating complex information quickly (…), consult with colleagues and seniors in a timely manner, (…)'containing' anxiety, and managing highly anxiety provoking situations (…) creating'good enough' collaboration with patients and families (…)” (Europe)	“… preparing trainees to gain skills in areas of emotional intelligence, compassionate training,… will assist them in meeting the challenge in the future” (Asia)
Specific interview skills	“… strange position of being experienced and inexperienced at the same time (…), I had to examine a 7-year-old boy in a playroom, I did not have a clue how to approach this. (…)” (Europe)	
Psycho-therapeutic skills	“What I think is a downside that we only study the basics of psychotherapy for half a year during our training. Since psychotherapy has a primary role among the therapeutic tools in child and adolescent psychiatry, I think that much more emphasis should be placed on teaching psychotherapy”. (Europe)	“There is an important disagreement… regarding the need for psychotherapeutic knowledge and its use in this field. Many, especially older ones agree it is of crucial importance because it represents a psychiatrist's main working domain, while others, especially younger ones build the core concept of their work on… the guidance of a therapeutic team around a patient and his/her family, of which a licensed psychotherapist (as a separate profession) is (or should be) an important part.” (Europe)
Key role of supervision	“Overall, it was a good experience… the safety of our patients because of lots of opportunities to discuss them with our mentors—trainers and… supervision” (Europe)	“It is not a matter of teaching technical skills but a true interaction that helps the therapeutic relationship between the trainee and the patient (through supervision…).” (Europe)
A professional identity combining both medicine and psychotherapy tradition	“It’s a very difficult situation with limited acknowledgment by other professions on the one hand, and clinical psychologists as well as regular psychiatrists working in the CAP field on the other (so why is there a CAP training when there are others invading the field?).” (Oceania)	
Work deeply grounded in a multi-disciplinary systems approach	“Another aspect of training that I appreciate is being part of true multi-disciplinary team working—I have learned so much from co-working cases with family therapists, art therapists and psychologists among others.” (Oceania)	“There is also little training in community psychiatry, there is no experience working with schools or parents during training.” (South-America)
Social-cultural dimension		
A strong influence of social-cultural aspects on mental health	“As one's experience of the world is tightly bound to one's cultural experience, mental health treatment should take this into account. (…) Ability to access such care may further be limited by cultural stigma towards mental health.” (North-America)	“I work and train doctors in an isolated area of the country… The experience makes the trainees some of the most skilled in the country in working with Indigenous youth. On the other hand, the trainees miss out on quality experiences in some of the more common… contexts…” (Australia)
Access to mental health service provision	“… to change is the stigma associated with seeking professional help for mental health difficulties, rather than relying on religious healers, which is a common approach by many” (Asia)	“National Health authorities… still are not convinced that child mental health needs to be one of their main concerns… All departments are in adult psychiatric hospitals and are considered by official managers as attached units” (Africa)

The first subtheme was related to *specific skills* that were seen as essential and specific for CAP training. Most related to the ability to contain a wide range of emotions and to reflect as essential for a psycho-therapeutic approach. Several trainees described how the specific interview skills required to build a therapeutic alliance with children and adolescents were rather new. Some styles of interview skills they had previously acquired during undergraduate medical training were perceived as less suitable for CAP practice. All the trainees who wrote about psychotherapy agreed that it was crucial for CAP training. Most trainers recognized that general medical training provides an essential basis for CAP practice, namely, rigor in diagnostic and therapeutic skills as well as learning to take responsibility in clinical practice. However, additional specific skills were mentioned, most of which were related to the need to create an appropriate relationship with patients and their families in CAP. The need for training in psychotherapeutic skills was stressed as essential for all CAP professional activities, which implies profound skill building throughout training. In depth, regular supervision was expected and stressed as essential for developing these skills by almost all trainee participants. Some trainees doubted whether psychotherapeutic skills could best be learned in a more conventional therapeutic context or should be acquired in a broader clinical work context closer to the future work-conditions of most CAP. Some trainers also mentioned limits to training these skills, mainly due to financial or other system factors that limit the amount of psychotherapy that trainees can conduct themselves.

Closely related, the professional identity of CAPs was described as being based on both a more general medical tradition and psychotherapy. Some experienced constraint of their role towards narrowed tasks for CAPs owing to high service demands and a re-distribution of tasks among other mental health professionals. The ability to work together in multi-disciplinary teams (both in and outside mental health care) was regarded as important, even more crucial in underserved areas with little or no CAP.

A final subtheme related to the importance of the *social-cultural dimension* of the biopsychosocial approach. It involved the influence of culture on mental health, as one's experience of the world is tightly bound to one's cultural experience, and that access to service provisions may be limited by cultural factors and stigma toward mental health.

### Theme 3: Trainers’ own and continuous professional development

Trainer: “Fortunately, we do have internal supervisor training to work on supervision skills.”

**Table 6:** Faculty development regarding training competencies

	Trainers’ quotations
Support and training needs for supervisors	
Training of supervising skills	“As a supervisor, we are very well supported by our state training committee. All of us undergo supervisor training and to remain accredited must attend regular supervisor debriefs and meetings, as well as pursue relevant professional developmental opportunities.” (Oceania)
Affiliation to a community of supervisors	*“*Despite the high workload with patients, I believe that we have a strong association of psychiatry with a strong subsection of CAP” (Europe)
An overall view making sense of the CAP training framework	
A reference to historical awareness and a long-term perspective	“Indonesia is a huge country with more than 250 million population, and 40% of its population consist of children and adolescents. By 2045, more than 70% of our total population will be in the productive age (15–64 years old)”. (Asia)
Political struggles to get CAP accepted as a specialty in its own rights	“Opposition and refusal of most of academic executives of adult psychiatry to admit child psychiatry as a separate specialty. National Health authorities were not and still are not convinced that child mental health needs to be one of main their concerns and deserve specific setting.” (Oceania)
Taking a broad whole systems-approach to address child mental health demands	“Child and adolescent mental health care needs to be approached as a wicked… complex, systemic, multi-causal, interconnected problem; needing collaborative input from multiple stakeholders with conflicting agendas; crossing organizational and disciplinary boundaries (…) These complex problems require multidimensional and integrated interventions that can only be provided within an integrated service model, connecting across contexts” (Oceania)
Influence on recruitment of CAP trainees	“We are looking for candidates able to connect emotionally with other people, who… collaborate with a wide range of professionals.” (Europe)
Frameworks of thinking to understand CAP trainees’ pathway	
Perceptions through both a more objective and a more subjective position	“This may require a paradigm shift for the trainee… the tension with the dominant biomedical paradigm (…) perhaps more oriented toward individualistic paradigms, and (…) diagnose and medicate. There is a need then to provide CAP trainees with a sound basis in thinking about and working with the person in their relational and psychosocial context and equipping them with reasoning skills that can support this way of working when confronted by an evidence base that may be individualistic, symptom focused and de-contextualized.” (Oceania)
	“They need to be trained not only as a CAP clinician (…), but also being an advocator…, case manager and collaborator with other mental health professionals to provide a better child and adolescent mental health system.” (Asia) “They need to develop self-awareness of their own psychological processes, in order to be able to use their emotions and emotional stability to let their patients grow.” (Europe)
The need to assume a paradigmatic shift for trainees during CAP training	“The training trajectory does not only make the trainees to transfer into a medical professional, but also often includes personal life-events, for instance having a child, becoming a parent.” (Europe)

Trainers were all very satisfied with their role as trainers. Most focused on the *help that they needed to develop supervisory skills*, to fulfill their roles and to provide appropriate training support through the experiential training of trainees. The mere possession of a high level of clinical skills was considered insufficient. A dedicated learning community of supervisors was mentioned as a valuable element to support each other, reflect together and improve supervision and training. A parallel was drawn with the essential role of coproduction and team support in daily clinical CAP practice.

As expected and consistent with their positions, most trainers presented a well-summarized *overall perspective* of CAP trainee pathways in their respective countries and the way training was organized within a larger societal scope. They highlighted that making sense of CAP training can be achieved only when an overarching view is taken, including the organization of (child) mental health services and public mental health issues. They referred to a growth perspective with past and ongoing changes in the system and provided examples of how challenges in these areas and potentially resolving these issues would influence CAP training and education in the near future. Some participants reported the recent recognition of CAP as a medical specialty in their countries and the effects of the perception of the CAP specialty on the recruitment of trainees. The responses related to this theme highlighted dynamic criticism, pointing out gaps and differing evolution alongside complex transformations of countries’ socioeconomic contexts and suggesting future improvements. They recognized an increasing public awareness of the importance of child and adolescent mental health and that to address the burning demands in child mental health, more was needed than mere CAP training but rather a wider systems approach.

Several *frameworks of thinking* emerged from the perspectives of trainers that allowed them to understand CAP trainees’ pathways and experiences. These seem to be situated along two orthogonal axes. On the one hand, CAP training is perceived from a more objective position, on the other hand from a more subjective position. The more objective position implies that trainers observe and measure progress of trainees with reliable indicators looking for patterns. The organization of training is done accordingly and aims to convey general knowledge and skills. The more subjective position focusses more on the individual experience of the trainee. It implies that knowledge is constructed by each individual trainee as they interact with their environment and with themselves. Through this framework trainers reported helping trainees develop a mindset or gestalt of a holistic, integrated approach to interacting with their patients and parents. Both frameworks were seen as valuable for CAP by trainers, yet learning how to shift flexibly along both axes for trainees was reported to be personally challenging and in the view of trainers needs to be supported.

## Discussion

Based on the indicators of perceived quality of training it appears that overall trainees and trainers are confident that the CAP training systems in their countries prepare trainees well for their future roles as child and adolescent psychiatrists. Variation is likely to reflect differences in systems of training and care between countries and regions across the world. The main goal of the present study was to understand *why* these differences exist and what we can learn from them. The thematic analysis of the international trainee’s and trainers’ perspectives and narratives emphasized the importance of the psychological security of the educational framework in CAP training: with a transparent, coherent curriculum; substantial social support and scaffolding to deal with contextual issues. They also highlighted a number of specific detailed skills needed for CAP and the importance of socio-cultural aspects for CAP. Trainers also emphasized the importance of support and training for supervisors, of a sense of coherence and overarching views for the training framework, and of flexibility between different frameworks of thinking to understand and guide trainees’ pathways.

The key role of a safe framework for supervision, with which both trainees and trainers agreed, seems highly consistent with the nature of post-graduate training. Internships in CAP are mainly based on clinical work experience from which the conceptualization of new knowledge emerges progressively, as described by Kolb’s model of experiential learning [23]. Supervision provides guidance and implies notions of tutorship, as described by Bedart, Frenay, Turgean and Paquay, where the tutor is a cognitive companion to the trainee [24]. Desirable, are regular meetings devoted to the trainee’s needs where learning starts from the contextual situation, and reflexivity is promoted through Socratic questioning. Supervisors adjust, as they are more present and directive at the beginning and gradually reduce and change their input, as the trainee makes progress in reflexivity and autonomy in learning. This is a good fit with continuous learning in medical education and with an interactive and reflexive training format aligned with adult learning strategies [25]. When such regular and reliable guidance is lacking, as several trainees reported in our study, they tend to experience an overwhelming level of autonomy that does not fit with their level of competence yet. Although work-experience with little supervision may promote critical thinking skills or facilitate learning later on in training, when there is not enough reflection on what is done correctly or incorrectly, trainees may just continue and fail to challenge or reflect upon their own bad practice. To make matters worse, some trainees experienced an absence of presence and guidance alongside high clinical demands. They described how their trainers were most willing to provide supervision but were unable to do so due to a lack of training resources and high clinical demand on the trainers. A few trainers reported similar situations, whereas others highlighted a lack of supervisory skills training.

One of the key specific skills mentioned was being able to contain a wide variety of emotions and other psycho-therapeutic skills. These skills are not limited to (child and adolescent) psychiatry, as many other medical specialties face situations where the emotions of their patients and team members need to be contained in a supportive way, e.g., in palliative care or emergency units [26]. However, the number of other, more technical skills that also play a part in those specialties may help to better regulate emotions by providing technical responses to suffering. In psychiatry, supporting emotional regulation and psychotherapeutic skills may at times be the most important response to patients and their caretakers feeling overwhelmed, desperate, and discouraged in the face of suffering. This implies the high value of continuing the tradition of psychiatry trainees being trained in the psychotherapies in addition to offering them more medical knowledge and skills [27].

The fact that the importance of specific skills training in a supportive and stimulating environment also applies to trainers themselves was emphasized by the perceived importance of faculty development to them. Educational skills for medical specialists are not innate, can be developed and are eminently teachable. Having these skills does not necessarily mean that trainers know how to teach what is most useful for their trainees in the best way and how to create optimal circumstances. For a long time, their own teachers were not necessarily the best role models for teaching practice, as trainers in medical education have based their teaching and supervision mainly on a trainer-centered approach, promoting teaching with lectures as the typical format [28]. Medical specialist postgraduate training is historically based on a master-apprentice model, where trainees learn on the job in clinical rotations guided by their more experienced trainers, who set goals for them at different stages during their training. Conversely, a learner-centered approach seems more in line with the learning needs highlighted by our trainee participants. This implies training that promotes reflexivity; co-construction of knowledge, skills and attitudes between trainers and trainees and formative assessment encapsulated in summative evaluations [29]. Despite the initial higher cost and time required at the beginning of training, flexible adjustments in supervision can be tailored to trainees’ needs during training. The current study highlighted that regional social, economic, and cultural issues potentially have a major influence on care provision and training quality but that individual or small group supervision and reflection could ensure a reliable educational framework despite the heavy clinical workload reported in several countries.

The importance of different frameworks of thinking reported by trainers resonates with the literature discussing the most appropriate models of medical education [30, 31]. In our study, the views of trainers argue for a comprehensive view that includes elements of both a more objective and a more subjective view of training and of trainees’ pathways. This suggests that, at least for (child and adolescent) psychiatry clinical practice and training, it is most productive to hold both these positions in mind. In such a view, the more objective framework allows for evidence-based education and enables trainees to develop rigor and a (neuro-) scientific approach in the field of psychiatry, which has been influenced by many opinions and judgments over a long history [29]. The more subjective framework protects against the risk of becoming reductionistic. It embraces the high complexity of daily clinical practice in psychiatry, acknowledging the subjective experience of each individual as they try to learn about the development of mental health. This framework shifts towards understanding CAP as part of mental health development, growth and resilience. Indeed, trainers highlighted the need to integrate multiple perspectives in medical specialist training not limited to trainees’ and trainers’ views but many others including patients, other physicians and health care professionals, faculty members, administrative managers, politicians, and, ultimately, society. Accordingly, some authors have supported the assumption of eclecticism and a complementary approach in methods and epistemologies in medical education research, such as complementary views of post-positivism and constructivism [30–32].

### Strengths and limitations

Despite its exploratory nature, the results from this attempt to collect experiences and analyze them via a qualitative approach triggered reflexivity about medical specialist training in different cultural contexts while suggesting opportunities to develop a few international standards in this area. Written responses to the internet-based survey allowed broad data collection with a wide reach across the globe.

However, the present study is based on a convenience sample of CAP trainees and trainers. It does not cover all countries with a CAP training program and in some countries such a program may still be absent or rudimentary organized, nor does it explore specific regional differences within countries. The collection method with written information about experiences and perspectives prevented interactivity that is part of other qualitative data collection; e.g., interviews or focus groups allow for reflection and interaction with and between participants in an iterative process. The thematic results in the present study could not be sent back to the participants for their comments because the initial responses were anonymized for ethical reasons, so further refinement through an additional triangulation process was not possible. All data was provided in English by mostly non-native speaking participants, and it may well be that this inhibited their expression or lead to some mis-understandings in data analysis and interpretation. While the personal clinical and training experience of all authors helped them to understand participants’ perspectives in depth, as insiders, they may have missed complementary views that could have been provided better by non-CAP and non-medical researchers e.g., with a background in health anthropology. Finally, the chosen methodology and qualitative approach may have led to a selection of participants and a bias of report towards more psychotherapy oriented aspects of training versus other relevant aspects including neuro-biological approaches to assessment and treatment.

## Conclusions

This study shows that, regardless of where postgraduate training in child and adolescent psychiatry takes place, a number of elements are important to prepare trainees for good care provision. Trainees need a standardized comprehensive and transparent curriculum; scaffolding to address their training paths at multiple levels that are adjusted to socioeconomic and cultural specificities; specific psychotherapeutic and interdisciplinary skills training; and enhanced and consistent supervision adjusted to their training needs. Trainers need to be offered opportunities for adjusted supervision skills training and to be embedded in a supportive supervisor community of practice. Helping them reflect on frameworks of thinking that form the basis of trainers’ perceptions of trainees’ training could ultimately enhance and diversify the quality of support provided to trainees. As these themes are so generic, increased international cooperation may help build cornerstones to reduce inequalities in national and international psychiatry training frameworks while respecting each cultural richness and context.

## Data Availability

Original data available upon request.
